# Smart Flexible Porous Bilayer for All‐Day Dynamic Passive Cooling

**DOI:** 10.1002/smsc.202300237

**Published:** 2024-01-10

**Authors:** Zuoxin Hu, Yu Qiu, Jicheng Zhou, Qing Li

**Affiliations:** ^1^ School of Energy Science and Engineering Central South University Changsha 410083 China

**Keywords:** all‐day dynamic passive cooling, evaporative cooling, hydrogels, polydimethylsiloxane, radiative cooling

## Abstract

Radiative cooling and evaporative cooling are sustainable cooling techniques without additional energy input. However, radiative cooling lacks dynamic cooling ability, while evaporative cooling demands external water replenishment, hindering their applications. Herein, a smart radiative/evaporative cooling bilayer combining a polydimethylsiloxane (PDMS) upper layer with a hydrogel lower layer is presented for efficient all‐day dynamic passive cooling. The PDMS layer with high solar reflectivity (0.930) and emissivity (0.952) provides excellent all‐day radiative cooling and protects the hydrogel from solar exposure, while the hydrogel layer demonstrates remarkable water evaporation and absorption, achieving dynamic evaporative cooling. Thus, the synergy of the two layers significantly enhances the overall cooling performance. Specifically, the bilayer can achieve the peak cooling power values of 424.4 and 650.6 W m^−2^ as well as the maximum subambient cooling temperatures of 10.4 and 3.7 °C during sunny and cloudy mid‐days, respectively. Moreover, the bilayer obtains 3.2 °C warmer temperature compared with the PDMS alone during cold nighttime, while the two structures exhibit comparable cooling performance during hot nighttime, indicating the self‐adaptive cooling property of the bilayer. In addition, the bilayer can achieve good cooling performance even under continuous cloudy days, offering a promising strategy for efficient all‐day dynamic passive cooling.

## Graphical abstract

A smart flexible porous bilayer that combines a porous polydimethylsiloxane (PDMS) upper layer and a hydrogel lower layer is designed for efficient all‐day dynamic passive cooling. The PDMS can reflect sunlight and radiate heat, while the hydrogel can regulate temperature by evaporating and absorbing water, enabling the bilayer to cool under high‐temperature conditions and retain warmth under low‐temperature conditions.

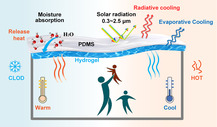

## 1 Introduction

Space cooling is essential and pervasive in modern society. However, present cooling equipment primarily relies on the vapor compression refrigeration cycle, which consumed ≈8% of the world's electricity and emitted 1.02 Gt CO_2_ in 2022.^[^ ^1^ ^]^ To reduce both the electricity consumption and carbon emission, passive cooling technologies like radiative cooling and evaporative cooling are emerging as viable alternatives.^[^ ^2^ ^]^

Radiative cooling that employs special structures to dissipate thermal radiation into the outer space through the atmospheric window (8–13 μm) can effectively lower the space temperature to levels below the ambient without any energy consumption.^[^ ^3^ ^]^ In 2013, Rephaeli et al. theoretically designed a metal–dielectric photonic structure that can achieve continuous daytime radiative cooling by tuning its spectral response.^[^ ^4^ ^]^ Subsequently, a nanophotonic cooler consisting of seven alternating dielectric layers atop a silver mirror was built and tested by Raman et al. in 2014, achieving a subambient cooling temperature of 4.9 °C.^[^ ^5^ ^]^ However, cost reduction and large‐scale deployment of such photonic structures remain challenging.^[^ ^6^ ^]^ Recent advancements in radiative cooling have led to the development of diverse structures, including multilayer structures,^[^ ^7^ ^]^ metamaterials,^[^ ^8^ ^]^ randomly distributed particle structures,^[^ ^9^ ^]^ and porous structures.^[^ ^10^ ^]^ For example, Zhai et al. fabricated a transparent metamaterial by randomly embedding micro‐SiO_2_ spheres into a polymethylpentene film, achieving a high total emissivity (*ε*_8–13 μm_) of 0.93 within the atmospheric window and a high cooling power value of 93 W m^−2^ when backed with a silver coating.^[^ ^11^ ^]^ Cheng et al. developed a coating with BaSO_4_ and SiO_2_ particles, achieving a high solar reflectivity (*R*_sun_) of 0.95% and a *ε*_8–13 μm_ of 0.96, thus leading to an average cooling power of 89.6 W m^−2^ and a maximum subambient cooling temperature of 8.1 °C at noon.^[^ ^12^ ^]^ Sun et al. proposed a cooling paper with the *R*_sun_ of 94% and the *ε*_8–13 μm_ of 0.95, obtaining subambient cooling temperatures of 6.0–8.8 °C.^[^ ^13^ ^]^ Additionally, radiative cooling technology has also been applied in building energy saving,^[^ ^14^ ^]^ atmospheric water harvesting,^[^ ^15^ ^]^ and personal thermal management.^[^ ^16^ ^]^

However, the maximum cooling power of most existing radiative cooling devices is limited to around 150 W m^−2^,^[^ ^17^ ^]^ which prevents their application in numerous practical scenarios that require higher cooling power. Moreover, most existing radiative cooling techniques were designed for a static cooling mode, disregarding the crucial need for aligning cooling power with demand. As a result, these techniques continue to cool even at night or under low‐temperature conditions, leading to undesired energy consumption for heating requirements.^[^ ^18^ ^]^ To overcome this challenge, dynamic radiative cooling layers made of specific phase‐change materials (VO_2_, etc.) have been proposed to regulate the emissivity in the atmospheric window, thereby adjusting the cooling performance.^[^ ^19^ ^]^ However, it is still quite hard to fabricate these layers due to their complex structures.^[^ ^19^ ^]^ Therefore, exploring novel approaches to improve cooling power and enable smart dynamic regulation of temperature in passive cooling technology is essential.

Evaporative cooling is another passive cooling technology that can absorb heat through water evaporation and release heat through water adsorption,^[^ ^20^ ^]^ thus regulating the space temperature dynamically.^[^ ^21^ ^]^ With cooling power exceeding 300 W m^−2^,^[^ ^22^ ^]^ evaporative cooling has been applied in diverse fields such as building cooling,^[^ ^23^ ^]^ data center cooling,^[^ ^24^ ^]^ and electronic device cooling.^[^ ^25^ ^]^ For example, Wang et al. designed a metal–organic framework with a high water‐absorption capacity over a wide humidity range, achieving a cooling power of 315 W m^−2^ and a passive cooling temperature of 14 °C below that of a photovoltaic panel.^[^ ^26^ ^]^ Sui et al. created a heat sink based on moisture desorption, maintaining a passive cooling temperature of 11.5 °C for around 400 min, thus enhancing the performance of an actual computing device by 32.65%.^[^ ^27^ ^]^

However, ensuring reliable water replenishment is a significant challenge for the widespread adoption of this technology, especially in water‐deficient regions.^[^ ^28^ ^]^ Moreover, the efficiency of evaporative cooling can be reduced under high‐humidity levels, as they reduce the water evaporation rate.^[^ ^29^ ^]^ Thus, developing and optimizing cooling technologies to overcome these challenges and enhance the efficacy of both evaporative and radiative cooling are crucial.

A combination of radiative cooling and evaporative cooling (i.e., radiative/evaporative cooling) is a promising strategy to overcome the challenges associated with each technology alone. Several studies have investigated the potential benefits of synergistic radiative/evaporative cooling to enhance cooling performance. Feng et al. presented a bilayer porous polymer film inspired by the cooling mechanism of human skin.^[^ ^30^ ^]^ This film consists of a hygroscopic hydrogel layer and a hydrophobic top layer with hierarchical pores, achieving a cooling power of ≈150 W m^−2^ and a maximum temperature difference of 7 °C. Li et al. developed a tandem radiative/evaporative cooling device by incorporating a hydrogel underlayer and a cellulose acetate fibrous network top layer.^[^ ^22^ ^]^ Their findings show that the average subambient cooling temperature of this tandem device can reach ≈10 °C, which is 8 °C lower than that achieved by radiative cooling alone. Then, Lu et al. introduced an insulation layer with low thermal conductivity (28 mW m^−1^ K^−1^) above the evaporative and radiative cooling layers, which blocked the heat transfer between the cooling space and the external environment.^[^ ^31^ ^]^ This device achieved a cooling power value of 96 W m^−2^ under direct sunlight and lowered the temperature by 9.3 °C compared to the ambient temperature. Furthermore, inspired by the fur layer of desert animals, Lu et al. developed an evaporation–insulation cooling system using hydrogels and aerogels, lowering ambient temperature by about 7 °C.^[^ ^32^ ^]^ Additionally, Xu et al. combined radiative and evaporative cooling functionalities within a single nanocomposite hydrogel, achieving a maximum temperature reduction of 6.2 °C under sunlight.^[^ ^33^ ^]^ Although some previous studies have explored the potential of combining radiative cooling and evaporative cooling, to the best of our knowledge, few existing cooling techniques have demonstrated the ability for dynamic passive cooling throughout the entire day.

In this article, a smart radiative/evaporative cooling bilayer combining a porous polydimethylsiloxane (PDMS) upper layer and a hydrogel lower layer is presented for efficient all‐day dynamic passive cooling. First, two scalable synthesis approaches were developed to fabricate the ultrawhite porous PDMS upper layer and the hydrogel lower layer, respectively. Subsequently, the micromorphology and optical properties of the obtained PDMS layer were analyzed and discussed. Additionally, the moisture absorption and evaporation of the hydrogel layer were investigated. Finally, the outdoor passive cooling performance of the bilayer on a sunny day and over 3 cloudy days was measured and evaluated. The novelty of this work lies in the development of a PDMS/hydrogel bilayer, which effectively combines the continuous radiative cooling capacity of PDMS with the dynamic water evaporation and adsorption capabilities of the hydrogel, thus achieving all‐day dynamic passive cooling. This design provides a novel and effective solution to dynamic passive cooling, further enhancing our understanding of radiative/evaporative cooling materials and systems.

## 2 General Concept

**Figure 1:**
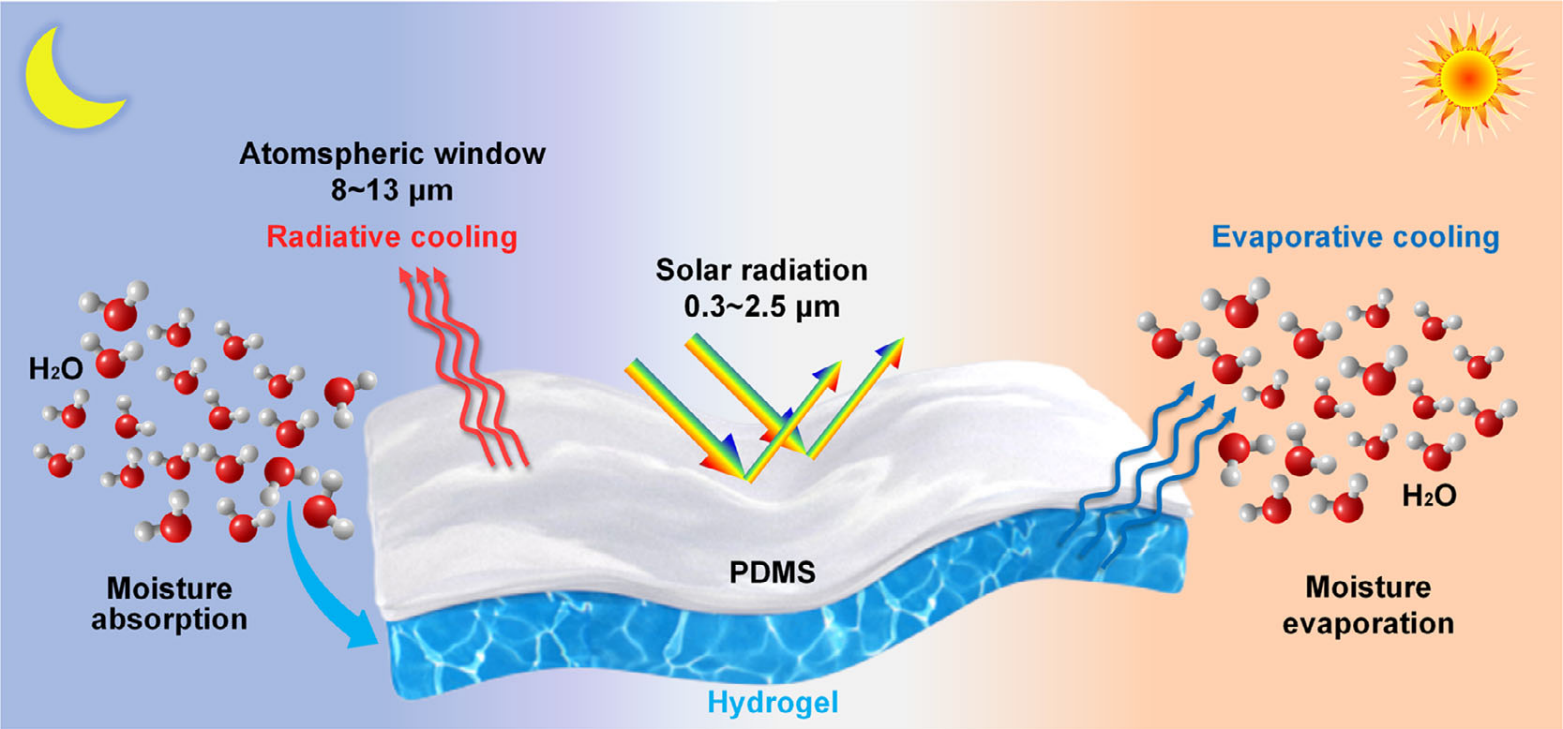
Design scheme and working principle of the smart flexible porous bilayer for all‐day dynamic passive cooling.

A bilayer consisting of an upper PDMS layer and a lower hydrogel layer is engineered to enable all‐day dynamic passive cooling. The design scheme and working principle of the bilayer are depicted in **Figure** **1**.

During the day, the PDMS layer with a hierarchically porous structure can strongly reflect sunlight due to its gradual refractive index transition at the polymer–air interface.^[^ ^34^ ^]^ Additionally, due to multiple molecular vibration modes, the PDMS layer is expected to achieve high total emissivity within the atmospheric window (8–13 μm), enabling excellent radiative cooling.^[^ ^35^ ^]^ Simultaneously, the hydrogel layer can release moisture to air by evaporation, enabling good evaporate cooling. Furthermore, the radiative cooling effect of the PDMS is helpful for maintaining relatively low hydrogel temperature, which is beneficial for moisture absorption and evaporative cooling. Additionally, the hydrophobic and porous PDMS not only allow moisture to permeate and infiltrate the hydrogel, but also can shield the hydrogel layer from solar irradiation, thereby preventing any solar energy absorption in the hydrogel.

At night, the hydrogel layer can effectively absorb and store atmosphere moisture, which can be utilized for evaporative cooling during the day or under high‐temperature conditions, thus enhancing the overall passive cooling capacity. Meanwhile, the hydrogel layer will release latent heat during the moisture absorption process at night, which can prevent the overcooling that usually occurs when only radiative cooling is employed.

To sum up, by combining radiative cooling and evaporative cooling, the bilayer is expected to achieve enhanced cooling power during the day or under high‐temperature conditions. Meanwhile, the hydrogel in the bilayer can prevent possible overcooling at night or under low‐temperature conditions. Consequently, the bilayer is expected to enable smart all‐day dynamic passive cooling.

## 3 Results and Discussion

### 3.1 Characterization of the Porous PDMS

The upper layer of the bilayer must facilitate moisture permeability and effective radiative cooling. White porous materials with nano‐ and microscale porous structures are typically ideal for this purpose. However, some existing methods have involved the use of hazardous chemicals, such as acetone, in the production of these materials, creating challenges for large‐scale production.^[^ ^36^ ^]^ Here, a porous white PDMS layer was fabricated by a sustainable and cost‐effective method based on NaCl template.

**Figure 2:**
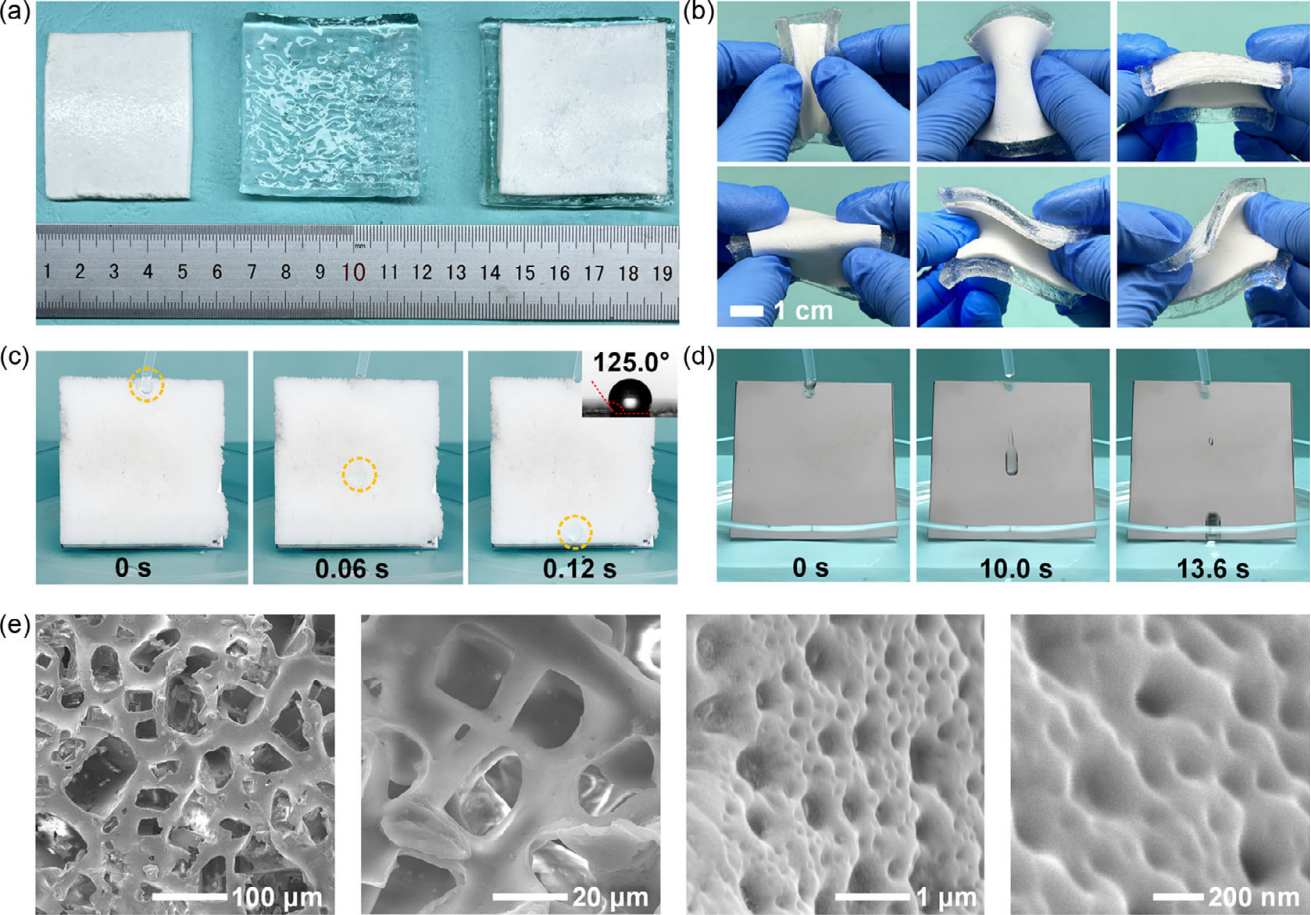
Structure and characteristics of the bilayer. a) Photographs of the PDMS, hydrogel, and bilayer. b) Photographs showing the excellent flexibility of the bilayer, which can easily recover its original shape after being twisted in various directions. c,d) Photographs of CuCl_2_ droplets sliding on the bilayer and an aluminum sheet, respectively, with an inset showing a water contact angle of 125.0° on the bilayer surface, indicating its hydrophobicity. e) SEM images of the PDMS.

The fabricated PDMS exhibits an ultrawhite appearance (see **Figure** **2**a). This characteristic serves as visual evidence of the PDMS's high reflectivity within the visible spectrum, which is beneficial for reflecting the incident sunlight. Furthermore, in practical applications, robust flexibility and mechanical integrity are crucial for passive cooling devices to be adaptable across diverse scenarios, including buildings, transport vehicles, and cold chains. Experiments have shown that the bilayer maintains structural integrity upon being folded and twisted in various directions (see Figure 2b), demonstrating its excellent flexibility and mechanical durability.

Additionally, to ensure optimal performance in rainy or high‐humidity environments, the passive cooling device must exhibit hydrophobic properties. The hydrophobicity of the PDMS layer was assessed by comparing the sliding behaviors of CuCl_2_ droplets on the PDMS and an aluminum sheet at a 60° tilt angle. As shown in Figure 2c, the CuCl_2_ droplet took 0.12 s to slide from the top to the bottom of the PDMS, leaving no residue. In contrast, a droplet took up to 13.56 s to slide from the top to the bottom of the aluminum sheet, with some liquid remaining on the sheet (see Figure 2d). This excellent hydrophobicity of the PDMS is further confirmed by its large water contact angle of 125.0° (see the inset in Figure 2c and S2, Supporting Information). The hydrophobic PDMS layer plays a crucial role in shielding the hydrogel layer from dust and dirt, ensuring that its moisture absorption and evaporation capabilities are preserved. These findings underline the remarkable mechanical durability and hydrophobic characteristics of the bilayer, making it well suited for diverse scenarios and outdoor applications.

Moreover, the microstructure of the PDMS is presented in Figure 2e and S3, Supporting Information, indicating that the PDMS has numerous hierarchical pores with pore sizes ranging from nanometer to micrometer scale. Notably, the porosity of the PDMS reaches 65.8%. These hierarchical pores not only can facilitate the permeation of moisture absorbed from the surrounding air into the hydrogel layer, but also can create gradient refractive index between the PDMS and air,^[10a]^ which is critical for improving both the solar reflectivity (*R*_sun_) and the total emissivity in the atmospheric window (*ε*_8–13 μm_).

**Figure 3:**
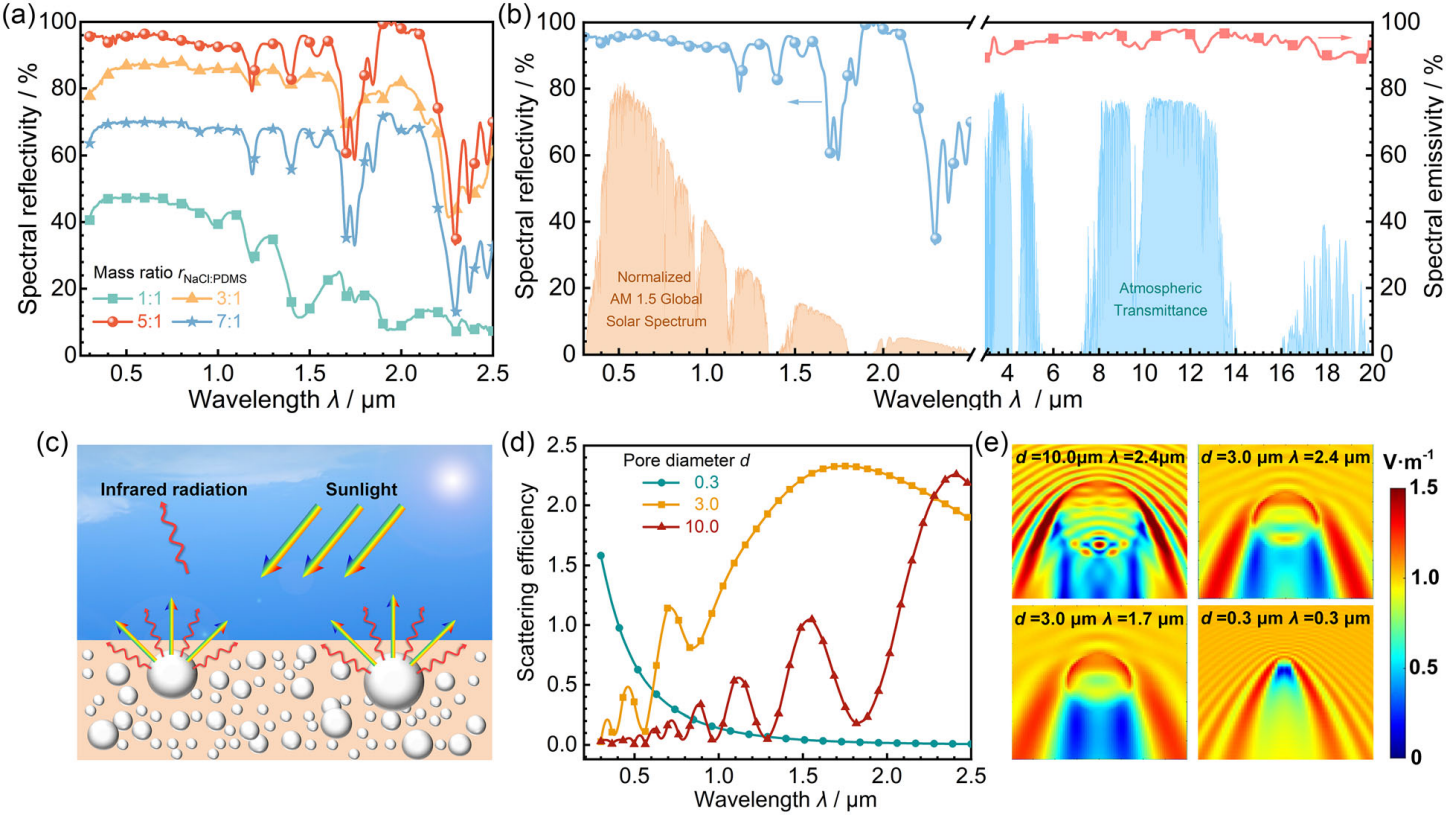
Optical characterization of the porous PDMS. a) Spectral reflectivity of a 2 mm‐thick porous PDMS with different mass ratios between the NaCl particles and the PDMS precursor (*r*_NaCl:PDMS_). b) Spectral reflectivity of the optimized PDMS in the solar band and its spectral emissivity in the atmospheric window band. c) Sketch of effective sunlight scattering by PDMS pores. d) Scattering efficiency of pores with different diameters in the PDMS at different wavelengths (*λ*). e) Electric field contours around pores with diameters of 0.3, 3.0, and 10.0 μm in the PDMS under different wavelengths (*λ*).

The radiative cooling performance of the PDMS is highly affected by the micromorphology of its hierarchical pores, which are directly influenced by the mass ratio between the NaCl particles and the PDMS precursor (*r*_NaCl:PDMS_). To optimize the mass ratio *r*_NaCl:PDMS_ for better radiative cooling performance, four mass ratios (i.e., 1:1, 3:1, 5:1, and 7:1) were tested. Figure S4, Supporting Information, shows that the surface of the PDMS changes from dense to loose as the mass ratio increases, indicating that adjusting the NaCl proportion can effectively adjust the porous structure of the PDMS. Reflectivity measurement shows that the spectral reflectivity of the PDMS increases significantly when *r*_NaCl:PDMS_ increases from 1:1 to 5:1 (see **Figure** **3**a). However, when *r*_NaCl:PDMS_ rises to 7:1, the spectral reflectivity decreases obviously. As a result, the solar reflectivity (*R*_sun_) of PDMS reaches its maximum value of 0.930 at the optimal *r*_NaCl:PDMS_ of 5:1. This high solar reflectivity can be attributed to the fact that this optimal NaCl proportion ensures a suitable porosity and pore size distribution in the PDMS, enhancing sunlight reflection.

Then, Figure 3b illustrates that the optimized PDMS can achieve high spectral reflectivity and high spectral emissivity within the main solar spectrum (*λ* = 0.3–2.5 μm) and the atmospheric window (*λ* = 8–13 μm), respectively. A high solar reflectivity (*R*_sun_) of 0.930 is achieved by the optimized PDMS. This is because the pores with different diameters in the PDMS can effectively reflect the electromagnetic waves in different wavelength (*λ*) bands. Finite‐difference time‐domain (FDTD) simulations confirm that the 0.3 μm pore can obtain the high scattering efficiency of above 1.0 when *λ* < 0.5 μm (see Figure 3d), while for the 3.0 μm pore and 10 μm pore, the corresponding *λ* ranges are mainly within 0.67–2.50 and 2.06–2.50 μm, respectively (see Figure 3d). Due to the cooperation of the scatterings introduced by the hierarchical pores within different bands, the PDMS can effectively scatter the sunlight across the main solar spectrum of 0.3–2.5 μm (see Figure 3c), which can significantly reduce the solar energy absorption and is a remarkable characteristic of the porous PDMS. The electric field contours in Figure 3e further highlight the significant scattering effects of the pores with different diameters on incident light across different wavelengths, supporting the PDMS's remarkable sunlight reflection ability. Moreover, the high solar reflectivity of the PDMS can also be explained by its complex refractive index. Because the PDMS has different complex refractive indexes in the visible–near‐infrared (vis–NIR) and midinfrared regions,^[^ ^37^ ^]^ in the vis–NIR region, the PDMS has a low real part and a near‐zero imaginary part of the refractive index, meaning it can absorb minimal sunlight but reflect the sunlight strongly.

Furthermore, when *λ* is within 3–20 μm, the spectral emissivity of the PDMS consistently exceeds 0.9 (see Figure 3b). As a result, it achieves a high total emissivity (*ε*_8–13 μm_) of 0.952 in the atmospheric window at 303 K. This is attributed to the polarized vibrations of PDMS molecules within the 8–13 μm band, which significantly improve spectral emissivity of the PDMS.^[^ ^37^ ^]^ In addition, the PDMS and air pores, which have high and low refractive indexes, respectively, can introduce sharp refractive index contrast at the PDMS–air interface, which is helpful for enhancing the light absorption/emission within the atmospheric window.^[^ ^38^ ^]^ These features facilitate the radiative heat exchange between the bilayer and the environment, resulting in the outstanding solar reflectivity and total emissivity that are expected to enable good radiative cooling.

### 3.2 Characterization of the Hydrogel

**Figure 4:**
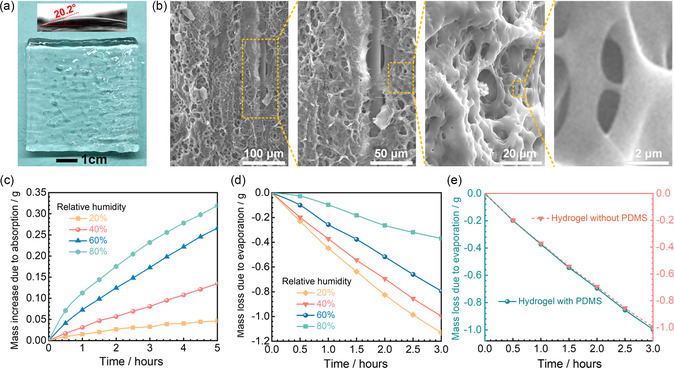
Structure and characteristics of the hydrogel. a) Photograph of the hydrogel, with an inset showing a water contact angle of 20.2°, indicating the hydrophilicity of the hydrogel. b) SEM images of the hydrogel. c,d) Mass change of the hydrogel due to absorption (at 15 °C) and evaporation (at 45 °C) under various RH levels. e) Mass loss curves due to evaporation (at 45 °C, 40% RH) for the hydrogel and the hydrogel with PDMS.

The fabricated lower hydrogel layer in the bilayer is a semitransparent flexible gel (see **Figure** **4**a). The hydrogel has numerous hierarchical micropores (see Figure 4b), which originate from the repeated freezing‐thawing process during the hydrogel preparation. This process leads to the formation of ice crystals, which in turn cause the densification of the dispersed components within the progressively diminishing volume between these crystals. Consequently, after the thawing and solvent exchange, the hydrogel exhibits a high density of micropores.^[^ ^39^ ^]^ These micropores serve as microchannels, facilitating moisture absorption and evaporation.

The moisture absorption capacity of the hydrogel is crucial for evaporative cooling. CaCl_2_, a widely used vapor adsorbent, was incorporated into the hydrogel to enhance the capacity of the bilayer.^[^ ^40^ ^]^ To evaluate this capacity, a 20 × 20 × 5 mm hydrogel sample was subjected to moisture absorption tests under various relative humidity (RH) levels at 15 °C. The experimental results demonstrate that the moisture absorption capacity of the hydrogel increases with rising RH at the same temperature (see Figure 4c). At 20% RH, the absorption capacity of the hydrogel reaches 23.1 g m^−2^ h^−1^, and this value reaches 67.5, 132.9, and 159.7 g m^−2^ h^−1^ when the RHs are 40%, 60%, and 80%, respectively. Notably, the maximum moisture absorption mass reaches 35.2% of the hydrogel's initial mass, indicating the hydrogel has a remarkable ability to self‐replenish its water from air, especially in high‐humidity environments. Additionally, the hydrophilicity of the hydrogel further demonstrates its moisture absorption ability, as confirmed by its small water contact angle (20.2°) in Figure 4a. Continuous advancements in atmospheric moisture harvesting materials offer the potential for further enhancing the moisture absorption capacity of the smart bilayer.^[^ ^41^ ^]^ This, in turn, can significantly improve its evaporative cooling performance.

The moisture evaporative capacity of the hydrogel is crucial for its application. Evaporation experiments were performed at 45 °C across different RH levels to assess this performance. The hydrogel was immersed in deionized water to fully swell before each experiment. Figure 4d shows that the moisture evaporation rate of the hydrogel increases with decreasing RH level. Specifically, in a 3 h experiment, evaporation rates of the hydrogel were 942.4, 830.8, 661.6, and 307.7 g m^−2^ h^−1^ at RH levels of 20%, 40%, 60%, and 80%, respectively. The hydrogel demonstrates a maximum moisture evaporation capacity of 42.95% of its initial mass, clearly indicating its excellent evaporative capacity under diverse environmental conditions.

If the hydrogel is irradiated by sunlight, the water in the hydrogel will strongly absorb sunlight, especially in the near‐infrared band,^[^ ^42^ ^]^ which is undesirable during cooling. To prevent sunlight from reaching the hydrogel, the hydrogel layer in the bilayer is covered by the PDMS layer with high solar reflectivity. Meanwhile, comparative evaporation tests of the hydrogels with and without the PDMS covering were conducted under 40% RH and 45 °C, finding that the mass loss of the hydrogel without the PDMS is 830.8 g m^−2^ h^−2^, and the value is 829.3 g m^−2^ h^−2^ for the hydrogel with the PDMS (Figure 4e). This negligible difference in the evaporative mass loss between the two samples indicates that the PDMS layer does not hinder the moisture evaporation from the hydrogel in the bilayer, which is essential for efficient evaporative cooling.

Most existing radiative cooling devices can only offer static cooling mode, which cannot meet the actual demand for dynamic cooling.^[^ ^43^ ^]^ Radiative cooling is beneficial during the daytime, but it may cause overcooling at night, thereby increasing heating demands.^[^ ^44^ ^]^ Hence, smart all‐day dynamic passive cooling technology that can adjust to various cooling and heating demands, and can operate efficiently is urgently needed. By evaporating and absorbing moisture under various environmental conditions, the fabricated hydrogel can exchange latent heat with the environment. During the day or under high‐temperature conditions, moisture evaporation from the hydrogel can cool its surroundings by absorbing latent heat.[^28^, ^45^] Conversely, at night or under low‐temperature conditions, the hydrogel can release latent heat to its surroundings when absorbing moisture from air, helping to keep the space warm.^[^ ^46^ ^]^ Therefore, the bilayer can leverage the properties of moisture evaporation and absorption to achieve all‐day dynamic passive cooling. This will be further confirmed in the outdoor passive cooling experiments.

### 3.3 Outdoor Passive Cooling Performance on a Sunny Day

**Figure 5:**
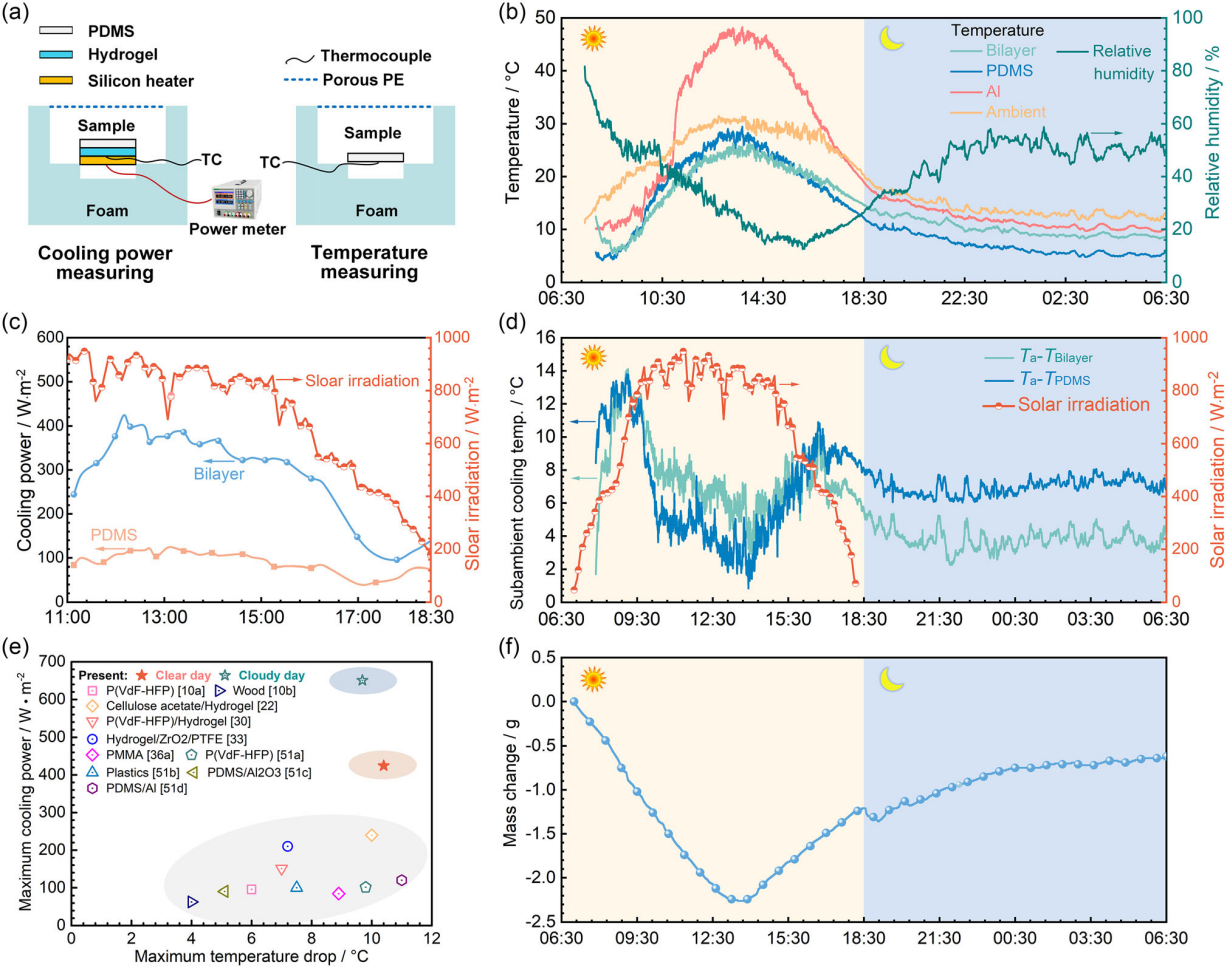
Passive cooling performance of the bilayer on a sunny day (March 5, 2023). a) Schematic of the outdoor testing device. b) Temperature change curves of the bilayer, PDMS, aluminum plate (Al), and environment; RH profile. c) Cooling power of the bilayer and PDMS at different solar irradiation levels. d) Solar irradiation and subambient cooling temperatures of the bilayer (*T*_a_–*T*_Bilayer_) and the PDMS (*T*_a_–*T*_PDMS_). e) Comparison of the passive cooling performance between the bilayer and some recently reported structures at noontime.^[^ ^10^, ^22^, ^30^, ^33^, ^36^, ^51^ ^]^ f) Mass change of the bilayer over the entire testing period.

A 24 h outdoor experiment was conducted on a sunny day (March 5, 2023) to comprehensively investigate the passive cooling performance of the bilayer. The sketches of the temperature and cooling power measurement devices are shown in **Figure** **5**a, and the photograph of outdoor measurement equipment can be seen in Figure S5, Supporting Information. The samples were individually placed inside insulated foam boxes covered with aluminum foil, with internal dimensions of 60 × 60 × 20 mm. The top surfaces of the foam boxes were covered with a 10 μm‐thick porous polyethylene membrane to reduce the effects of wind and allow moisture diffusion. PDMS and aluminum plate (Al) were used as comparison groups, and the bilayer was regarded as the experimental group. The cooling power of each sample was measured using a heating compensation method,^[10a]^ which is described in Text S2, Supporting Information.

Figure 5b,d shows the temperature data and ambient parameters recorded throughout the entire 24‐hour experimental period. During the whole day, Figure 5b demonstrates that the temperatures of both the bilayer and PDMS maintain lower than the ambient temperature (*T*_a_) across various humidity levels. During the high‐solar‐irradiation and high‐temperature period within 10:00–14:00, the average solar irradiation (*I*_avg_) and average ambient temperature (*T*_a,avg_) can reach 874 W m^−2^ (Figure 5d) and 28.0 °C (Figure 5b), respectively. Within this period, the subambient cooling temperature of the bilayer (*T*_a_–*T*_Bilayer_) ranges from 3.3 to 10.4 °C, reaching the average value of 6.9 °C, while the corresponding average value for the PDMS is just 4.2 °C (Figure 5d). In addition, further temperature comparison of different samples finds that the Al temperature, representing the actual temperature of the roof surface, can reach the maximum value of 48.3 °C at around 13:40, which is 17.1, 18.9, and 22.7 °C higher than the temperatures of environment, the PDMS, and the bilayer, respectively.

Moreover, when there is no solar irradiation from 18:30 to 6:30, the ambient temperature is as low as 11.6–20.0 °C with the average value of just 14.0 °C (Figure 5b). Under this condition, the temperature of the PDMS is as low as 4.8–12.0 °C (Figure 5b), which is 6.1–8.5 °C lower than the ambient temperature (Figure 5d). It is obvious that undesirable overcooling occurs under the low‐temperature condition without solar irradiation when only the PDMS is employed. However, the bilayer consistently demonstrates a higher temperature (8.2–14.7 °C, Figure 5b) than the PDMS, which is just 2.4–5.9 °C lower than the ambient temperature (Figure 5d). It can be found that the bilayer can obtain the average subambient cooling temperature of as low as 3.9 °C (Figure 5d), which is 3.2 °C (45%) lower than that of the PDMS (7.1 °C), indicating that the bilayer can effectively relieve the overcooling introduced by the PDMS.

To sum up, the developed bilayer can adjust its passive cooling ability dynamically. It not only can achieve satisfactory cooling performance during the day under high solar irradiation and high temperature but also can relieve the overcooling at night under low‐temperature condition without solar irradiation. This all‐day dynamic passive cooling performance is attributed to the cooperation between the upper PDMS layer and the lower hydrogel layer. During the day, the PDMS can both reflect the incident solar irradiation to the environment and emit infrared radiation to the outer space. Meanwhile, the water in the hydrogel will also take away heat from the bilayer through evaporation. For example, the bilayer continuously loses weight from 7:00 to 13:46 with a net mass loss of 2.26 g (Figure 5f). As a result, it can consistently exhibit higher cooling power compared to PDMS (Figure 5c). It can be found that the PDMS shows the maximum radiative cooling power of 122.4 W m^−2^ and the average power of 85.0 W m^−2^, which are consistent with some previous studies.^[^ ^47^ ^]^ However, the bilayer can achieve the maximum cooling power of up to 424.4 W m^−2^, which greatly exceeds that of the PDMS with radiative cooling alone. In the evening, the radiative cooling of the PDMS still works. However, the hydrogel starts to release heat through absorbing moisture (Figure 5f) when the RH goes up at night (Figure 5b), which is beneficial for improving the bilayer temperature and can prevent overcooling. The hydrogel can achieve such strong water absorption ability because it is loaded with CaCl_2_, a low‐cost vapor adsorbent.

In addition, the cooling performance of the bilayer is compared with some previously designed cooling structures, as presented in Figure 5e and Table S1, Supporting Information. At noontime (10:00–14:00), the bilayer can achieve the largest subambient cooling temperature and the peak cooling power of 10.4 °C and 424.4 W m^−2^, respectively. It can be found from Figure 5e that the subambient cooling temperature obtained by the bilayer is higher than the best value reported in most of the listed literature, and the cooling power is 184.4 W m^−2^ higher than the best value in the listed literature, indicating that the present bilayer design is a promising alternative for passive cooling.

### 3.4 Outdoor Passive Cooling Performance Over Three Cloudy Days

Clouds can significantly reduce atmospheric transmittance, which may reduce the radiative cooling performance significantly. Therefore, in this section, the passive cooling performance of the bilayer was evaluated during 3 continuous cloudy days from July 27, 2023, 5:30 to July 30, 2023, 5:30, and the effects of clouds were analyzed.

**Figure 6:**
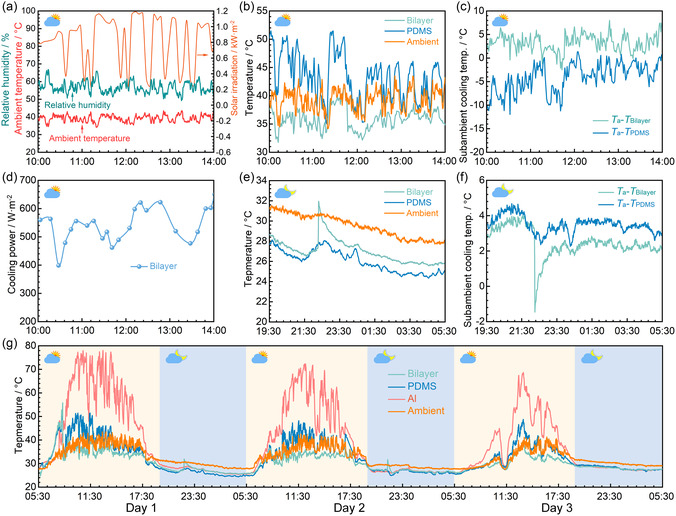
Passive cooling performance of the bilayer during 3 consecutive cloudy days. a) Ambient temperature, RH, and solar irradiation within 10:00–14:00 on the first cloudy day (27 July, 2023, similarly hereafter). b) Temperature change curves of the bilayer, PDMS, and environment within 10:00–14:00 on the first cloudy day. c) Subambient cooling temperatures of the bilayer (*T*_a_–*T*_Bilayer_) and the PDMS (*T*_a_–*T*_PDMS_) within 10:00–14:00 on the first cloudy day. d) Cooling power of the bilayer within 10:00–14:00 on the first cloudy day. e) Temperature change curves of the bilayer, PDMS, and environment within 19:30–05:30 on the first cloudy day. f) Subambient cooling temperatures of the bilayer (*T*_a_–*T*_Bilayer_) and PDMS (*T*_a_–*T*_PDMS_) within 19:30–05:30 on the first cloudy day. g) Temperature profiles of all samples and the environment over 3 consecutive cloudy days (from July 27, 2023, 5:30 to July 30, 2023, 5:30).

First, the performance indices of the bilayer and the PDMS were compared within 10:00–14:00 on the first cloudy day (27 July, 2023, similarly hereafter). It can be observed in **Figure** **6**a that the solar irradiation varies strongly within 224–1190 W m^−2^ with the average value (*I*_avg_) of 823 W m^−2^, and the average ambient temperature (*T*_a,avg_)/RH reaches 39.2 °C/46.0%. Temperature tests of the samples show that the PDMS temperature (35.1–51.5 °C) is generally higher than the ambient temperature (Figure 6b). The maximum difference between the ambient temperature and the PDMS temperature can reach −12.9 °C, and the corresponding average value is −4.1 °C (Figure 6c), indicating that PDMS cannot realize subambient cooling during the cloudy day because its radiative cooling performance is strongly weakened by clouds. In contrast, the bilayer temperature ranges from 31.8 to 41.4 °C (Figure 6b), and the corresponding maximum/average subambient cooling temperature reaches 9.7 °C/3.3 °C (Figure 6c), indicating that the present bilayer can still achieve good passive cooling. This is because the hydrogel in the bilayer provides strong cooling power while evaporating water into the air. As a result, the cooling power of the bilayer can reach 650.6 W m^−2^ with the average value of 539.5 W m^−2^ within 10:00–14:00 on this cloudy day (Figure 6d). Moreover, it is important that the bilayer can be averagely 7.4 °C cooler than the PDMS, successfully covering the shortage of the PDMS (Figure 6b,c).

Then, the temperatures of different samples are also compared in the evening within 19:30–5:30 on the first cloudy day, finding that the average temperatures of the environment, the PDMS, and the bilayer are 29.5, 26.0, and 27.0 °C, respectively (Figure 6e). It can be found that the average subambient cooling temperatures of the PDMS and the bilayer are 3.5 and 2.5 °C (Figure 6f), respectively, where the difference is just 1 °C, indicating they can achieve comparable cooling performance. These results signify that the bilayer can achieve satisfactory all‐day passive cooling performance on the cloudy day.

In addition, the temperatures of the bilayer and PDMS were continuously measured during 3 cloudy days (Figure 6g), and corresponding ambient parameters are shown in Figure S6, Supporting Information. During the daytime period with high solar irradiation (10:00–14:00), the bilayer consistently exhibits superior cooling performance compared with the PDMS. For the bilayer, its average subambient cooling temperatures within 10:00–14:00 reach 3.3, 3.9, and 1.1 °C during the 3 days, respectively. While the PDMS cannot realize subambient cooling most of the time, the average temperatures of the PDMS within 10:00–14:00 are 4.1, 3.5, and 3.5 °C higher than corresponding average ambient temperatures. During the night (19:30–5:30) without solar irradiation, both structures exhibit good passive cooling performance, and the average subambient cooling temperatures of the bilayer/PDMS within 19:30–5:30 are 2.5 °C/3.5 °C, 1.7 °C/2.2 °C, 2.1 °C/1.9 °C, respectively, during the 3 days. It can be found that the subambient cooling temperatures of the bilayer and PDMS are quite close in each day, indicating that the cooling performances of the two structures are comparable. The above results indicate that the present bilayer design can achieve continuous passive cooling even under cloudy conditions.

## 4 Conclusion

In summary, this study designs a smart flexible porous PDMS/hydrogel bilayer for all‐day dynamic passive cooling. Characterization of the bilayer indicates that its porous PDMS layer can achieve a high solar reflectivity of 0.930 within 0.3–2.5 μm and a high total emissivity of 0.952 within 8–13 μm. Meanwhile, its hydrogel layer possesses exceptional moisture evaporation and absorption performance, which enables efficient evaporative cooling and self‐replenishment of water. Moreover, an outdoor passive cooling experiment during a sunny day demonstrates that the bilayer can achieve efficient cooling performance with a peak power of 424.4 W m^−2^ and a maximum subambient cooling temperature of up to 10.4 °C between 10:00 and 14:00 under high solar irradiation and high temperature. At night, under low‐temperature conditions without solar irradiation, the bilayer can obtain 1.09–3.96 °C higher temperature than the PDMS, which effectively prevents overcooling. These results demonstrate the smart all‐day dynamic passive cooling ability of the bilayer. Additionally, a continuous passive cooling experiment conducted over 3 cloudy days reveals that the bilayer demonstrates superior cooling performance compared to the PDMS during the daytime. Specifically, it can achieve an average subambient cooling temperature of 3.7 °C within 10:00–14:00 of the 3 days, and its cooling power even reaches 650.6 W m^−2^ due to the excellent evaporative cooling. Meanwhile, both the bilayer and PDMS show comparable cooling performance at night, with the cooling temperature difference of only 0.2–1.0 °C. These results demonstrate that the bilayer can achieve continuous passive cooling even under cloudy conditions. Results from this work can provide an inspiration for dynamic passive cooling design in different weathers, which is expected to benefit a broad range of applications, such as energy‐saving buildings, solar cell cooling, and personal thermal management, thereby saving global energy consumption.

## 5 Experimental Section

5.1

5.1.1

##### Fabrication of the Porous PDMS

**Figure 7:**
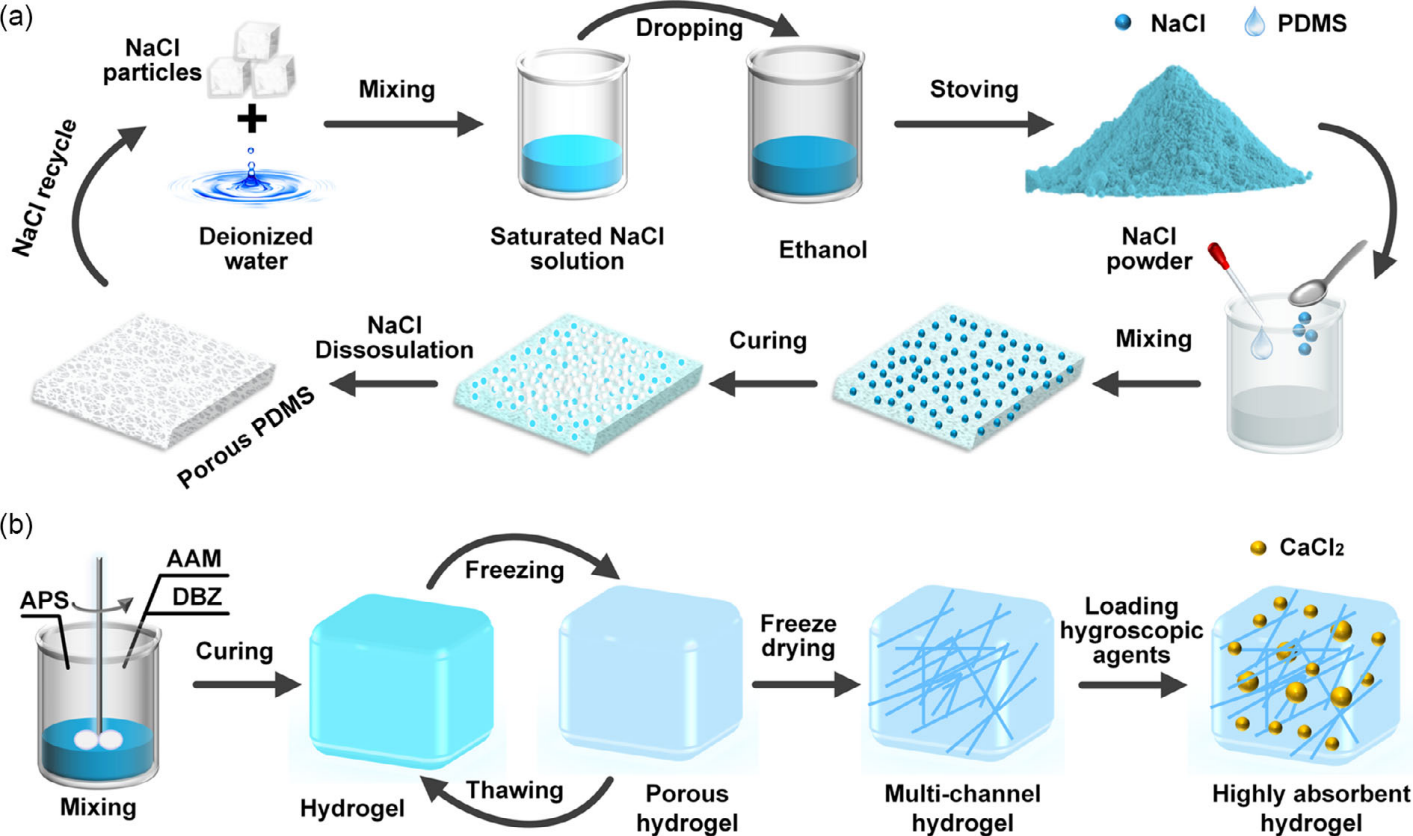
a) Schematic of the fabrication process of the porous PDMS using the NaCl template method. b) Schematic of the hydrogel fabrication process, which involves gelation, repeated freezing and thawing, freeze drying, and loading of moisture absorbent.

A sustainable and cost‐effective method based on NaCl template was used to fabricate the porous PDMS, as depicted in **Figure** **7**a. First, a saturated NaCl solution was prepared by mixing large‐sized NaCl particles (purchased from Xilong Scientific Co., Ltd.) with deionized water. This solution was then slowly dripped into 98.8% pure ethanol (purchased from Beijing Innochem Science & Technology Co., Ltd.) while being vigorously stirred. After 5 min, the solution was allowed to settle for 10 min at least, and then the upper layer of solution was removed. The resulting white NaCl precipitate was then collected and dried in an oven at 70 °C for 12 h to obtain NaCl powder with small particle size. Then, the PDMS prepolymer (Sylgard 184 A, purchased from Dow Global Corporation) and curing agent (Sylgard 184 B, purchased from Dow Global Corporation) were mixed with a mass ratio of 10:1 to prepare PDMS precursor. Then, a uniform blend was formed by adding an appropriate amount of NaCl powder to the PDMS precursor. The mass ratio of the PDMS precursor to NaCl powder was 1: *x* (*x* = 1, 3, 5, 7). The mixture was then poured into a mold and cured at 120 °C for 12 h, followed by immersion in deionized water at 60 °C for 48 h. After drying at 60 °C for 12 h, the resulting PDMS layers were obtained. During the above fabrication process, NaCl can be recycled.

##### Fabrication of the Hydrogel

The hydrogel was prepared through a continuous process involving gelation, repeated freezing and thawing, freeze drying, and loading of moisture absorbent, as shown in Figure 7b. First, a solution of 2 mol L^−1^ acrylamide (AAM) and 0.004 mol L^−1^ ammonium persulphate (APS) was stirred using a magnetic stirrer for 30 min. Next, 0.02 mol L^−1^ divinylbenzene (DBZ) was added as a crosslinking agent to this solution and stirred for 2 h. The solution was then allowed to stand for 10 h and cured in a thermostat at 70 °C for 2 h. The resulting hydrogel was subsequently washed repeatedly using deionized water at room temperature. AAM, APS, and DBZ were purchased from Aladdin Reagent Co., Ltd. Then, to impart moisture‐absorbing capabilities to the hydrogel, it was freeze‐dried to create a multichannel structure. The hydrogel was then frozen at −45 °C for 8 h and thawed in deionized water at room temperature. This freezing‐thawing process was repeated three times. Then, after being freeze‐dried at −45 °C for 12 h, the hydrogel was then immersed in a 50% mass fraction CaCl_2_ (purchased from Sinopharm Chemical Reagent Co., Ltd.) aqueous solution to improve its moisture absorption properties. Finally, the PDMS and hydrogel were assembled to form the bilayer (see Figure 2a).

##### Material Characterization

The solar reflectivity of the PDMS within 0.3–2.5 μm was measured using a UV–vis–NIR spectrophotometer (UV‐3600i PLUS, Shimadzu) equipped with an integrating sphere (ISR‐603, Shimadzu). The midinfrared reflectivity of the PDMS within 3–20 μm was measured using a Fourier transform infrared spectrometer (Nicolet Nexus670, Thermo Fisher). The microstructures of the PDMS and hydrogel were obtained by a scanning electron microscope (SEM, JSM‐IT300LA, JEOL). The radiative cooling performance of the PDMS mainly depends on its optical properties in the solar and midinfrared bands. The solar reflectivity (*R*_sun_) can be calculated using Equation (1). The total emissivity of the PDMS at 303 K in the atmospheric window of 8–13 μm (*ε*_8–13 μm_) can be calculated using Equation (2).

(1) $$R_{\text{sun}}=\frac{\int_{0.3\;\text{μm}}^{2.5\;\text{μm}}R_{\lambda }I_{\text{AM}1.5}(\lambda )d\lambda }{\int_{0.3\;\text{μm}}^{2.5\;\text{μm}}I_{\text{AM}1.5}(\lambda )d\lambda }$$

(2) $$\varepsilon_{8–13\;\text{μm}}=\frac{\int_{8\;\text{μm}}^{13\;\text{μm}}\varepsilon_{\lambda }I_{\text{BB}}(\lambda )d\lambda }{\int_{8\;\text{μm}}^{13\;\text{μm}}I_{\text{BB}}(\lambda )d\lambda }\;\;\;,\;\;\;\;\varepsilon_{\lambda }=1-R_{\lambda }$$

where *R*_*λ*_ signifies the spectral reflectivity of the sample; *I*_AM1.5_(*λ*) corresponds to the AM1.5 solar irradiation spectrum; *ε*_*λ*_ is the spectral emissivity of the sample; and *I*_BB_(*λ*) is the spectral emissive power of blackbody at 303 K.

##### Numerical Simulation

Optical simulations were performed using ANSYS Lumerical 2020 based on the FDTD method to calculate the scattering efficiency of incident light (0.3–2.5 μm) by various pores within the porous PDMS (see Figure S1, Supporting Information).^[^ ^48^ ^]^ The incident light was modeled as a total‐field scattered‐field source, and total field scattering field boundary conditions were applied in the simulation calculations. The electromagnetic field distributions were obtained by frequency domain field profile monitors. The refractive index of PDMS in solar wavelengths was ≈1.42.^[^ ^49^ ^]^

##### Outdoor Experiments on Passive Cooling Performance

Outdoor experiments were conducted on both a sunny day and across three consecutive cloudy days on the rooftop of a building at Central South University, Changsha, China (28.1736°N, 112.9368°E). The solar irradiation was measured with a pyranometer (RS‐TBQ‐N01‐AL, Shandong Renke), while the temperature and humidity of the environment were recorded with a thermohygrometer (COS‐03‐5, Shandong Renke). The temperatures of samples were measured with thermocouples (TT‐K‐36‐SLE, Omega) and monitored using a data acquisition module (DAM‐3039, Beijing Art Technology). An electronic balance (SP601, OHAUS) was used to record the mass change of the bilayer. The cooling power was determined by heating a silicon wafer at the bottom of the sample using an electric power meter (SS‐6010KD, Dongguan Bufan Electronics).

##### Statistical Analysis

The spectral reflectivity data were directly obtained using previously mentioned instruments, and OrigionPro software was used to plot the obtained data without any further processing. The temperature, RH, mass, solar irradiation, and water contact angle were directly measured, and their uncertainties were provided by the manufacturers, as shown in Table S2, Supporting Information. The passive cooling power of the sample (*P*_cool_) was indirectly measured, and its uncertainty was analyzed by using the quadratic power method proposed by Kline.^[^ ^50^ ^]^ A detailed description of the uncertainty analysis is presented in Text S3, Supporting Information. *P*_cool_ can be expressed as Equation (3), and the absolute uncertainty of *P*_cool_ (Δ*P*_cool_) can be calculated using Equation (4). Detailed Δ*P*_cool_ data of all *P*_cool_ results are presented in Table S3, Supporting Information, where the Δ*P*_cool_ values were within 0.82–13.21 W m^−2^. The relative uncertainty of *P*_cool_ ($\varepsilon_{P_{\text{cool}}}$) can be calculated using Equation (5), which is found to be just 2.03%.

(3) $$P_{\text{cool}}=\frac{P_{\text{heat}}}{S}$$

(4) $$\begin{array}{c} \Delta P_{\text{cool}}={\left[{\left(\frac{\partial P_{\text{cool}}}{\partial P_{\text{heat}}}\right)}^{2}{\left(\Delta P_{\text{heat}}\right)}^{2}+{\left(\frac{\partial P_{\text{cool}}}{\partial S}\right)}^{2}{\left(\Delta S\right)}^{2}\right]}^{\frac{1}{2}}\\ \;={\left[{\left(\frac{1}{S}\right)}^{2}{\left(\Delta P_{\text{heat}}\right)}^{2}+{\left(-\frac{P_{\text{heat}}}{S^{2}}\right)}^{2}{\left(\Delta S\right)}^{2}\right]}^{\frac{1}{2}} \end{array}$$

(5) $$\varepsilon_{P_{\text{cool}}}=\frac{\Delta P_{\text{cool}}}{P_{\text{cool}}}$$

where *P*_heat_ is the total heating power of the silicon wafer, W; Δ*P*_heat_ is the absolute uncertainty of *P*_heat_, W m^−2^; *S* is the area of the silicon wafer, m^2^; and Δ*S* is the absolute uncertainty of *S*, which is evaluated in Text S3, Supporting Information.

## Conflict of Interest

The authors declare no conflict of interest.

## Supporting information

Supplementary Material

## Data Availability

The data that support the findings of this study are available from the corresponding author upon reasonable request.
