# Supplementary material for: Smart Flexible Porous Bilayer for All‐Day Dynamic Passive Cooling
Source: Small Sci. 2024 Jan 10;4(3):2300237. doi: 10.1002/smsc.202300237 (PMC11935011; doi:10.1002/smsc.202300237)
Supplement: Supplementary file 1 — Supplementary Material [file SMSC-4-2300237-s001.pdf]

Supporting Information

A Smart Porous Bilayer Polymer for All-Day Dynamic Passive Cooling

Zuoxin Hu, Yu Qiu, Jicheng Zhou, Qing Li**

School of Energy Science and Engineering, Central South University, Changsha, 410083, China

*Corresponding authors
E-mail addresses: yu.qiu@csu.edu.cn (Y. Qiu); qingli@csu.edu.cn (Q. Li)

This PDF file includes:
Text S1 to S3
Figs. S1 to S6
Table S1 to S3
References

Contents

Text S1. Theoretical calculation of passive cooling power 3
Text S2. Measurement of passive cooling power using the heat compensation method 4
Text S3. Uncertainty analysis 5
Supplementary Figures 7
Fig. S1 Schematic of the simulation domain. 7
Fig. S2 Measurement of water contact angle at different regions of the PDMS. 8
Fig. S3 SEM images of the PDMS. 9
Fig. S4 Photographs of the PDMS prepared with different mass ratios between the NaCl particles and PDMS precursor ($r_{NaCl:PDMS}$). 10
Fig. S5 Photograph of the outdoor testing platform. 11
Fig. S6 Ambient temperature, relative humidity, and solar irradiation during the cooling performance measurement in three continuous cloudy days. 12
Table S1. Comparison of passive cooling performance 13
Table S2. Information of instruments 14
Table S3. Results of uncertainty analysis of cooling power 15
References 17

Text S1. Theoretical calculation of passive cooling power

The net cooling power of the upper PDMS layer through radiative cooling ($P_{\mathrm{rad}}^{\mathrm{net}}$) can be calculated by using **Equation (S1)**.[1]

$$P_{\mathrm{rad}}^{\mathrm{net}} = P_{\mathrm{rad}} - P_{\mathrm{env}} - P_{\mathrm{solar}} \quad \text{(S1)}$$

where P_{rad} is the radiative power from the PDMS to the outer space; P_{env} is the power absorbed from the environment through radiative, convective and conductive heat transfer; P_{solar} is the absorbed solar irradiation power on the PDMS surface.

The evaporative cooling power of the hydrogel (P_{eva}) can be calculated by using **Equation (S2)**.[2]

$$P_{\mathrm{eva}} = \frac{\Delta H \cdot \Delta m}{t \cdot A} \quad \text{(S2)}$$

where ΔH is the enthalpy of water vaporization; Δm represents the weight loss of the hydrogel; t denotes the evaporation time; A represents the evaporation area of the hydrogel.

Then, the passive cooling power of the bilayer (P_{cool}) can be calculated by using **Equation (S3)**.

$$\begin{aligned} P_{\mathrm{cool}} &= P_{\mathrm{rad}}^{\mathrm{net}} + P_{\mathrm{eva}} \\ &= P_{\mathrm{rad}} - P_{\mathrm{env}} - P_{\mathrm{solar}} + P_{\mathrm{eva}} \end{aligned} \quad \text{(S3)}$$

Text S2. Measurement of passive cooling power using the heat compensation method

The passive cooling power of the bilayer or the PDMS layer (P_{cool}) is measured by using a widely-used heat compensation method.[3]

In this method, a silicon wafer is placed below the sample, and a thermocouple is inserted between the wafer and the sample to measure the sample temperature. If the sample temperature remains below the ambient temperature throughout the measurement process, it indicates that the passive cooling has been achieved.

During the test, a direct-current (DC) power supply is used to heat the silicon wafer to make the sample temperature be the same as the ambient temperature. At this condition, the total heating power (P_{heat}) measured by the power meter in the DC power supply is equal to the total passive cooling power of the sample.

Text S3. Uncertainty analysis

An uncertainty analysis is necessary to ensure the reliability of the experiment.

In present experiment, the temperature, relative humidity, mass, solar irradiation, water contact angle were directly measured, and their uncertainties were provided by the manufacturers, as shown in **Table S2**.

Only the silicon wafer area (S) and the passive cooling power of the bilayer or the PDMS (P_{cool}) were indirectly measured, and their uncertainties were analyzed by using the quadratic power method proposed by Kline.[4] In this method, firstly, the absolute uncertainty (ΔY) of a dependent variable Y can be calculated by using **Equation (S4)**. Then, the relative uncertainty of the dependent variable (ε_Y) can be expressed as **Equation (S5)**.

$$\Delta Y = \left[\left(\frac{\partial Y}{\partial x_1}\right)^2 \left(\Delta x_1\right)^2 + \left(\frac{\partial Y}{\partial x_2}\right)^2 \left(\Delta x_2\right)^2 + \ldots + \left(\frac{\partial Y}{\partial x_n}\right)^2 \left(\Delta x_n\right)^2\right]^{\frac{1}{2}} \tag{S4}$$

$$\varepsilon_Y = \frac{\Delta Y}{Y} \tag{S5}$$

where Y is a variable dependent on n independent variables, x_n signifies the n independent variables of the function; Δx is the absolute uncertainty of the variable x.

Based on this method, firstly, the absolute uncertainty (ΔS) of the area (S) of the silicon wafer is analyzed. S can be expressed as the product of the length (a) and width (b) of the wafer, as shown in **Equation (S6)**. Because a=0.04000.0005 m and b=0.04000.0005 m, ΔS can be calculated by using **Equation (S7)**.

$$S = ab \tag{S6}$$

$$\begin{aligned} \Delta S &= \left[b^2\left(\Delta a\right)^2 + a^2\left(\Delta b\right)^2\right]^{\frac{1}{2}} \\ &= \left[0.0400^2 \times \left(0.0005\right)^2 + 0.0400^2 \times \left(0.0005\right)^2\right]^{\frac{1}{2}} \\ &= 2.828 \times 10^{-5}\ \mathrm{m}^2 \end{aligned} \tag{S7}$$

Then, the passive cooling power of the sample (P_{cool}) can be expressed as **Equation (S8)**. The absolute uncertainty of P_{cool} (ΔP_{cool}) can be calculated by using **Equation (S9)**. Detailed ΔP_{cool} data of all P_{cool} results are shown in **Table S3**, where the ΔP_{cool} values are within 0.82~13.21 $\mathrm{W \cdot m^{-2}}$. The relative uncertainty of P_{cool} (ε_{Pcool}) can be calculated by using **Equation (S10)**, which is found to be

just 2.03%, confirming the reliability of the experimental results.

$$P_{\text{cool}} = \frac{P_{\text{heat}}}{S} \tag{S8}$$

$$\Delta P_{\text{cool}} = \left[\left(\frac{\partial P_{\text{cool}}}{\partial P_{\text{heat}}} \right)^2 \left(\Delta P_{\text{heat}} \right)^2 + \left(\frac{\partial P_{\text{cool}}}{\partial S} \right)^2 \left(\Delta S \right)^2 \right]^{\frac{1}{2}} \tag{S9}$$

$$\begin{aligned} \varepsilon_{P_{\text{cool}}} &= \frac{\Delta P_{\text{cool}}}{P_{\text{cool}}} = \left[\left(\frac{\partial P_{\text{cool}}}{\partial P_{\text{heat}}} \right)^2 \left(\frac{\Delta P_{\text{heat}}}{P_{\text{cool}}} \right)^2 + \left(\frac{\partial P_{\text{cool}}}{\partial S} \right)^2 \left(\frac{\Delta S}{P_{\text{cool}}} \right)^2 \right]^{\frac{1}{2}} \\ &= \left[\left(\frac{1}{S} \right)^2 \left(\frac{\Delta P_{\text{heat}}}{P_{\text{cool}}} \right)^2 + \left(-\frac{P_{\text{heat}}}{S^2} \right)^2 \left(\frac{\Delta S}{P_{\text{cool}}} \right)^2 \right]^{\frac{1}{2}} \\ &= \left[\left(\frac{1}{0.0016} \right)^2 \left(0.01 \right)^2 + \left(-\frac{1}{0.0016^2} \right)^2 \left(\frac{2.828 \times 10^{-5}}{1} \right)^2 \right]^{\frac{1}{2}} \\ &= 2.03\% \end{aligned} \tag{S10}$$

where P_{heat} is the total heating power measured by the power meter; S is the area of the silicon wafer.

Supplementary Figures

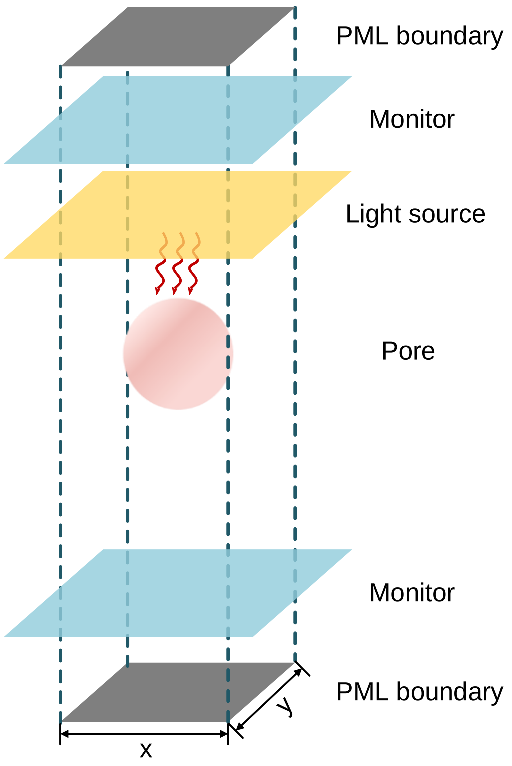

Fig. S1 Schematic of the simulation domain.

We established a three-dimensional simulation domain and simulated the transports of normally incident electromagnetic waves with different wavelengths. **Fig. S1** shows the schematic of the simulation domain. The Finite-Different Time-Domain (FDTD) method was used to simulate the transports of electromagnetic waves by solving the Maxwell's equations expressed in Equations. (S11)-(S14).[5]

$$\nabla \times \boldsymbol{H} = \boldsymbol{J} + \frac{\partial \boldsymbol{D}}{\partial \tau} \tag{S11}$$

$$\nabla \times \boldsymbol{E} = -\frac{\partial \boldsymbol{B}}{\partial \tau} \tag{S12}$$

$$\nabla \cdot \boldsymbol{B} = 0 \tag{S13}$$

$$\nabla \cdot \boldsymbol{D} = \rho \tag{S14}$$

where $\boldsymbol{H}$ is magnetic field; $\boldsymbol{E}$ is electric field; $\boldsymbol{J}$ is electric current density; ρ is charge density; $\boldsymbol{D}$ is electric displacement field; $\boldsymbol{B}$ is magnetizing field.

We used ANSYS Lumerical 2020 to perform the FDTD simulation and calculate the scattering efficiency.[6] The scattering efficiency is the ratio between the scattering cross-section area and the geometry area of an object.

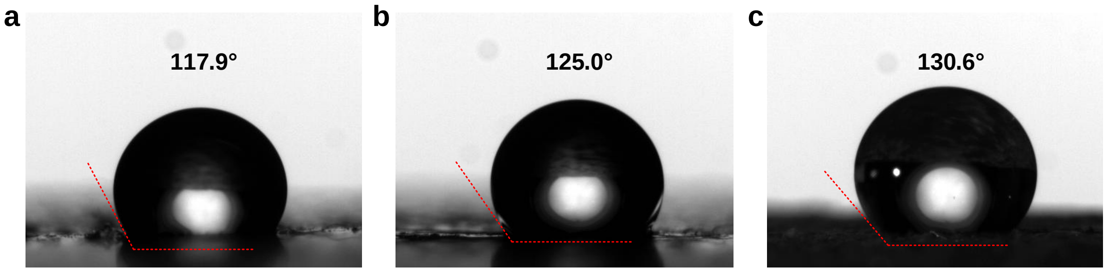

Fig. S2 Measurement of water contact angle at different regions of the PDMS.

The designed PDMS exhibits excellent hydrophobicity, as demonstrated by the measurement of its water contact angle, shown in **Fig. S2**. The water contact angle ranges from 117.9° to 130.6° at three different regions of the PDMS surface, and the 125.0° can be selected as the representative value for PDMS.

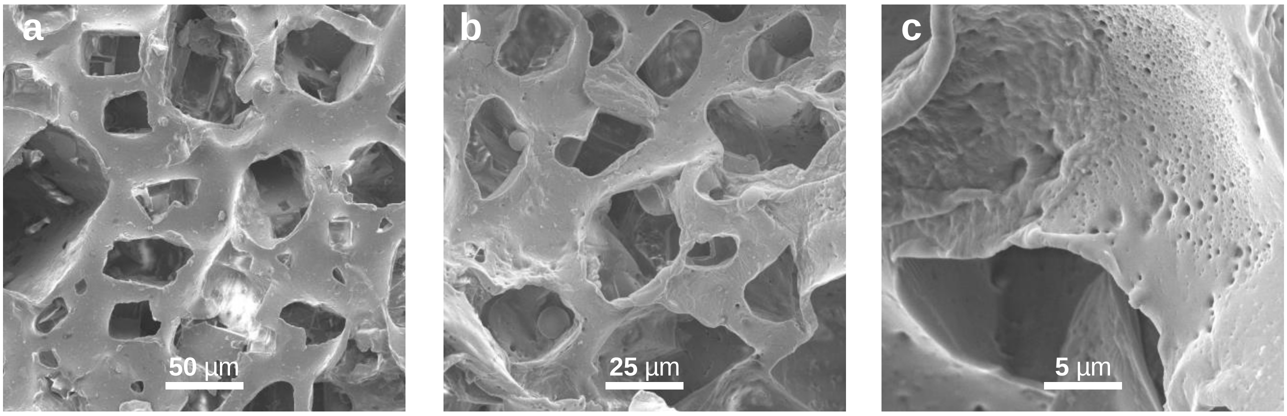

Fig. S3 SEM images of the PDMS.

Fig. S3 shows the scanning electron micrograph (SEM) of the PDMS. The SEM images reveal that the PDMS has many porous structures with a size distribution ranging from nanometers and micrometers.

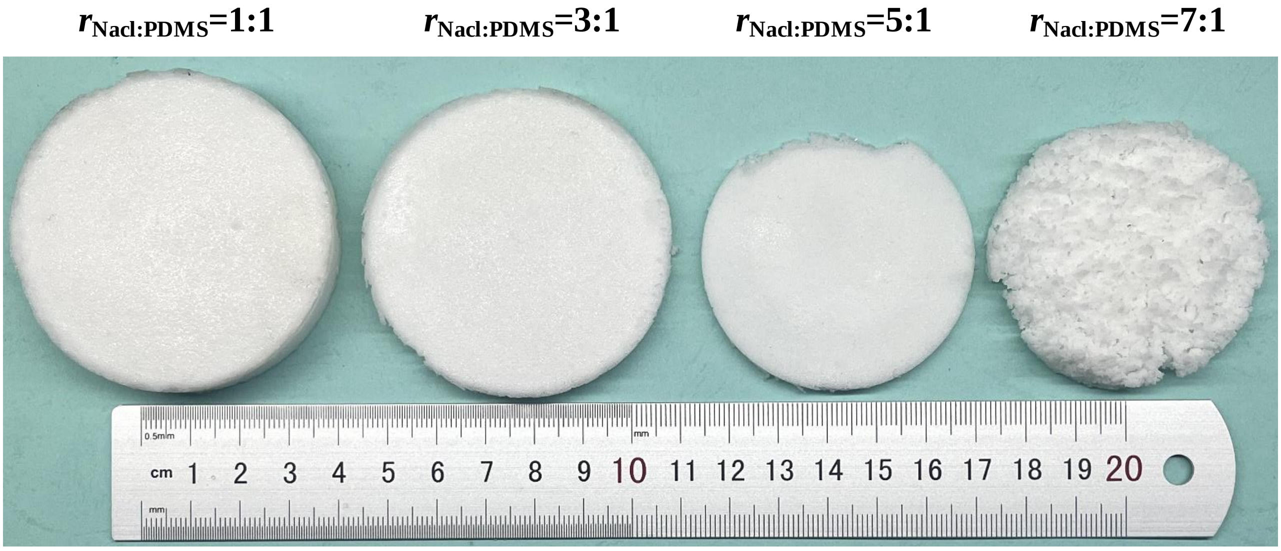

Fig. S4 Photographs of the PDMS prepared with different mass ratios between the NaCl particles and PDMS precursor ($r_{NaCl:PDMS}$).

The reflectivity of PDMS is dependent on the mass ratio between the NaCl particles and PDMS precursor ($r_{NaCl:PDMS}$). We conducted a study on the spectral reflectivity of the PDMS to determine the optimal ratio for improving reflectivity. **Fig. S4** presents the photographs of fabricated PDMS using various mass ratios. As the mass ratio increases, it can be seen in **Fig. S4** that the surface of the PDMS shifts from dense to loose, indicating that the proportionof NaCl particles affects the surface morphology of the PDMS obviously. The reflectivity measurements indicate that the maximum reflectivity is achieved when $r_{NaCl:PDMS}$ equals to 5:1.

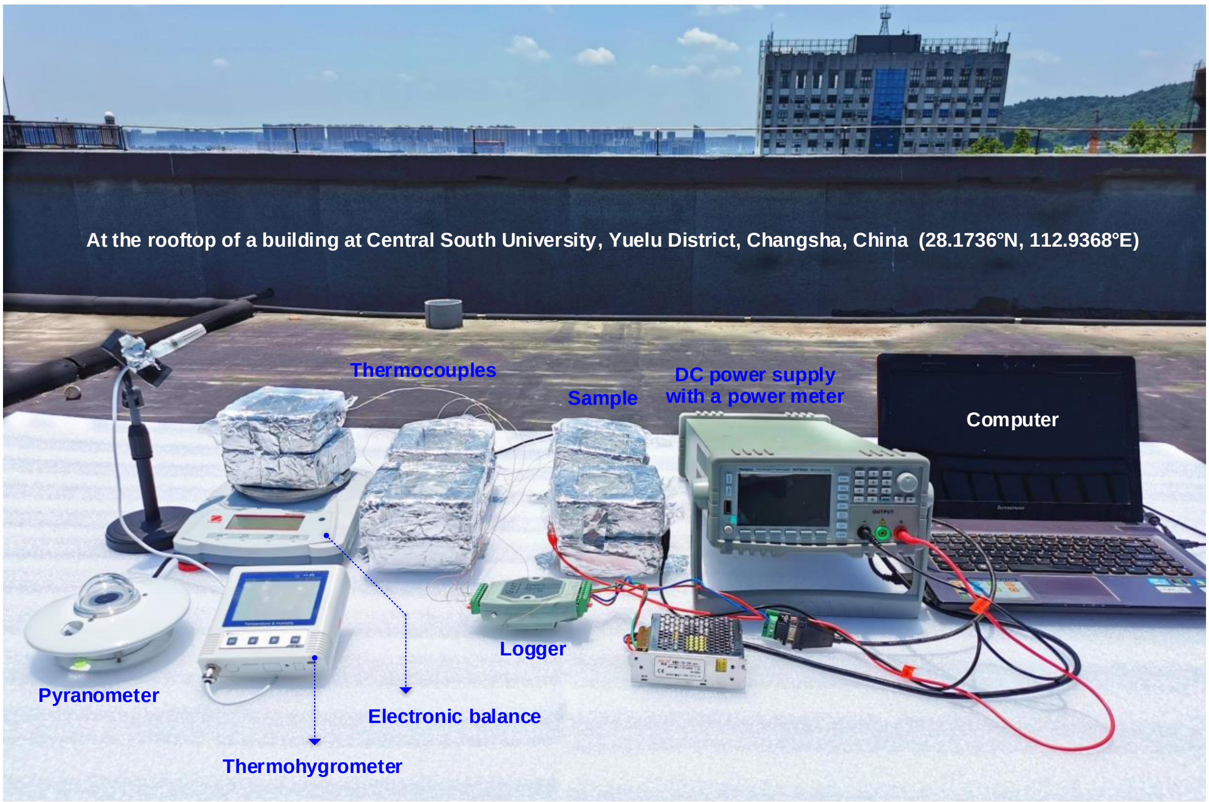

Fig. S5 Photograph of the outdoor testing platform.

The outdoor passive cooling performance testing platform is shown in **Fig. S5**. The equipment in the platform can be divided into two parts, including the temperature measurement part and the cooling power measurement part.

The temperature measurement part consists of thermocouples (TT-K-36-SLE, Omega), a data logger (DAM-3039, Beijing Art Technology), and a computer (IdeaPad Y470, Lenovo). The cooling power measurement part consists of a silicon wafer heater and a DC power supply with a power meter (SS-6010KD, Dongguan Bufan Electronics). The ambient temperature and relative humidity (RH) were measured by a thermohygrometer (COS-03-5, Shandong Renke), and the solar irradiation of incident sunlight was measured by a pyranometer (RS-TBQ-N01-AL, Shandong Renke). All devices are placed on a table that is 750 mm above the rooftop of a building. A 5 cm-thick white foam layer is placed on the table to ensure that the platform is not strongly heated by sunlight.

Outdoor experiments were conducted on both a sunny day (March 5, 2023) and across three continuous cloudy days (July 27, 2023, 5:30 to July 30, 2023, 5:30) on the rooftop of a building in Central South University, Changsha, China (28.1736°N, 112.9368°E).

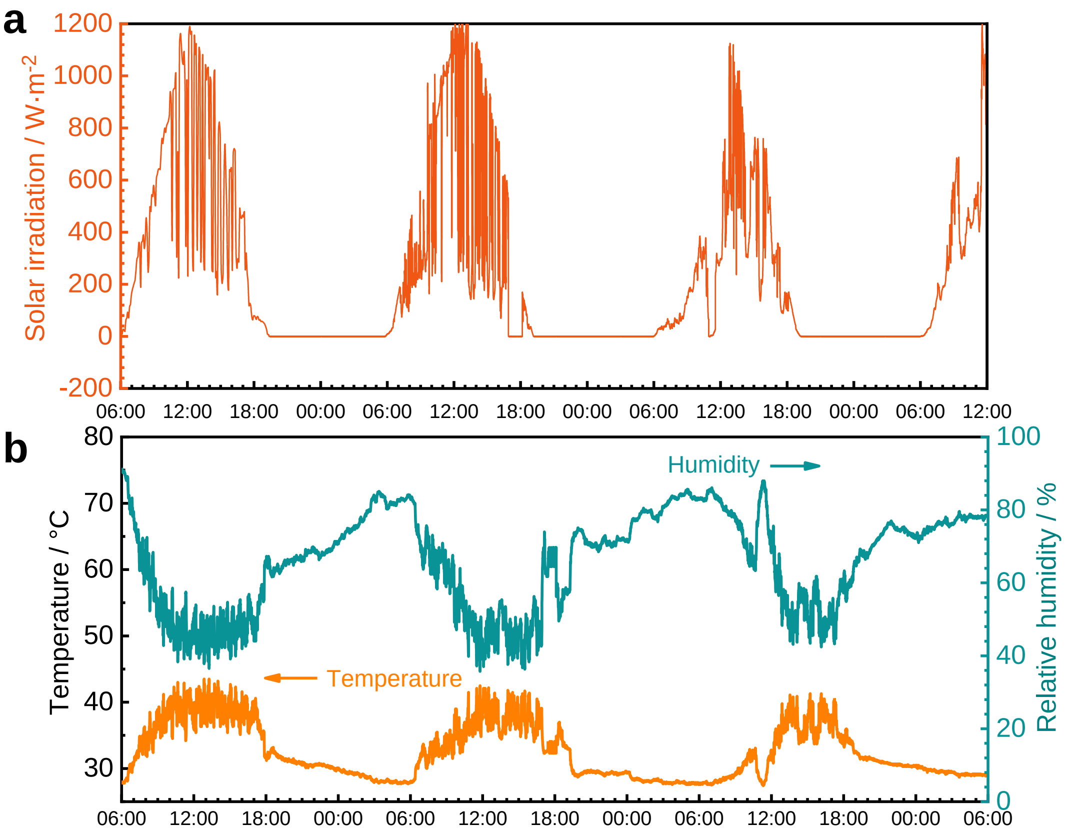

Fig. S6 Ambient temperature, relative humidity, and solar irradiation during the cooling performance measurement in three continuous cloudy days.

Table S1. Comparison of passive cooling performance

We compared the passive cooling performance of the designed bilayer with some published passive cooling structures in **Table S1**. These passive cooling structures include double-layer structures that combine radiative cooling and evaporative cooling, as well as individual radiative cooling structures. The comparison parameters include solar reflectivity (R_{sun}) and total emissivity in atmospheric window of 8~13μm ($\varepsilon_{8\text{-}13\mu m}$), maximum subambient cooling temperature, and the maximum cooling power ($P_{cool,max}$).

Table S1 Performance comparisons among the bilayer polymer and some previous cooling structures.

**Structure**	**Materials**	R_{sun} / $\varepsilon_{8\text{-}13\mu m}$	**Solar irradiation (W·m^{-2})**	**Maximum subambient cooling temperature (°C)**	$P_{cool,max}$ **(W·m^{-2})**	**Year**	**Ref.**
**This work**	**PDMS/hydrogel**	**0.930/0.952**	**874.4**<br>**823.0**	**10.4**<br>**9.7**	**424.4**<br>**650.6**	**2023**	/
Sponge	P(VdF-HFP)	0.945/0.956	916.7	9.8	101.5	2023	[7]
Bilayer polymer	Cellulose acetate/Hydrogel	0.950/0.940	~980	10	240	2022	[3]
Nanocom-posite	Hydrogel/ZrO_2/ PTFE	0.890/0.900	~700	7.2	210	2022	[2a]
Nano-Micro-Structure	Plastics	0.960/0.900	975	7.5	100	2021	[8]
Bilayer polymer	P(VdF-HFP)/Hydrogel	0.960/0.960	~800	7	151	2021	[9]
Porous structure	PMMA	0.950/0.980	860	8.9	85	2020	[10]
Photonic structure	PDMS/Al_2O_3	0.950/0.960	862	5.1	90.8	2020	[11]
Porous structure	Wood	0.960/-	~750	~4	63	2019	[12]
Multilayer structure	PDMS/Al	0.900/0.946	~853.5	11	120	2019	[13]
Hierarchical pores	P(VdF-HFP)	0.960/0.970	890	~6	96	2018	[14]

Table S2. Information of instruments

Table S2 Information of instruments

Instrument	Model number	Manufacturer	Origin Country	Measurement range	Uncertainty
UV-VIS-NIR Spectrometer	UV-3600iPLUS	Shimadzu	Japan	0.2~2.5 μm	/
Fourier Infrared Spectrometer	Nicolet Nexus670	Thermo Fisher	America	4000~400 cm^{-1}	/
Scanning Electron Microscope	JSM-IT300LA	JEOL	Japan	/	3.0 nm
Mercury Porosimeter	AutoPore IV 9500	Microelectronics Instrument	China	50 A~11000 μm	0.11%
Contact Angle Meter	SL200KB	KINO	America	0~180°	0.1°
Pyranometer	RS-TBQ-N01-AL	Jianda Renke	China	0~2000 $W·m^{-1}$	1 $W·m^{-1}$
Thermohygrometer	COS-03-5	Jianda Renke	China	-40~+80 ℃<br>0~100% RH	0.1 ℃<br>1.5% RH
Thermocouple	TT-K-36-SLE	TT-K-36-SLE	America	-200~260 ℃	0.1 ℃
Electronic Balance	SP601	OHAUS	America	0~600 g	1 mg
Power Meter	SS-6010KD	Bufan Electronics	China	0~600 W	1%
Ruler	Dl8020	Deli	China	0~200 mm	0.5 mm

Table S3. Results of uncertainty analysis of cooling power

Table S3 Results of uncertainty analysis of cooling power

Total heating power (W)	Cooling power ($W \cdot m^{-2}$)	Absolute uncertainty ($W \cdot m^{-2}$)	Relative uncertainty
0.391	244.40	4.96	2.03%
0.465	290.80	5.90	2.03%
0.505	315.60	6.41	2.03%
0.531	331.60	6.73	2.03%
0.602	376.40	7.64	2.03%
0.679	424.40	8.62	2.03%
0.637	398.40	8.09	2.03%
0.641	400.80	8.14	2.03%
0.582	363.60	7.38	2.03%
0.600	374.80	7.61	2.03%
0.603	376.80	7.65	2.03%
0.619	386.80	7.85	2.03%
0.618	386.00	7.84	2.03%
0.580	362.80	7.36	2.03%
0.573	358.40	7.28	2.03%
0.588	367.20	7.45	2.03%
0.586	366.40	7.44	2.03%
0.536	335.20	6.80	2.03%
0.516	322.40	6.54	2.03%
0.524	327.60	6.65	2.03%
0.516	322.40	6.54	2.03%
0.522	326.00	6.62	2.03%
0.508	317.60	6.45	2.03%
0.469	293.20	5.95	2.03%
0.449	280.40	5.69	2.03%
0.430	268.80	5.46	2.03%
0.236	147.60	3.00	2.03%
0.171	106.80	2.17	2.03%
0.153	95.60	1.94	2.03%
0.191	119.20	2.42	2.03%
0.256	160.00	3.25	2.03%
0.083	51.60	1.05	2.03%
0.120	75.20	1.53	2.03%
0.114	71.20	1.45	2.03%
0.131	82.00	1.66	2.03%
0.065	40.40	0.82	2.03%
0.152	94.80	1.92	2.03%
0.108	67.20	1.36	2.03%
0.109	68.00	1.38	2.03%

0.129	80.40	1.63	2.03%
0.134	84.00	1.71	2.03%
0.896	560.00	11.37	2.03%
0.901	563.13	11.43	2.03%
0.638	398.75	8.09	2.03%
0.766	478.75	9.72	2.03%
0.842	526.25	10.68	2.03%
0.889	555.63	11.28	2.03%
0.864	540.00	10.96	2.03%
0.890	556.25	11.29	2.03%
0.791	494.38	10.04	2.03%
0.828	517.50	10.51	2.03%
0.739	461.88	9.38	2.03%
0.783	489.38	9.93	2.03%
0.850	531.25	10.78	2.03%
0.957	598.13	12.14	2.03%
0.994	621.25	12.61	2.03%
0.950	593.75	12.05	2.03%
0.996	622.50	12.64	2.03%
0.832	520.00	10.56	2.03%
0.763	476.88	9.68	2.03%
0.828	517.50	10.51	2.03%
0.960	600.00	12.18	2.03%
0.964	602.50	12.23	2.03%
1.041	650.63	13.21	2.03%

References

[1] a) Y. Zhai, Y. Ma, S. N.David, D. Zhao, R. Lou, G. Tan, R. Yang, X. Yin, *Science* **2017**, 355, 1062; b) T. Du, J. Niu, L. Wang, J. Bai, S. Wang, S. Li, Y. Fan, *ACS Appl. Mater. Interfaces* **2022**, 14, 51351; c) D. Zhao, A. Aili, Y. Zhai, S. Xu, G. Tan, X. Yin, R. Yang, *APPL PHYS REV* **2019**, 6, 021306.

[2] a) L. Xu, D. W. Sun, Y. Tian, L. Sun, T. Fan, Z. Zhu, *ACS Appl. Mater. Interfaces* **2022**, 14, 45788; b) R. Li, Y. Shi, M. Wu, S. Hong, P. Wang, *Nat. Sustain.* **2020**, 3, 636; c) C. Wang, L. Hua, H. Yan, B. Li, Y. Tu, R. Wang, *Joule* **2020**, 4, 435.

[3] J. Li, X. Wang, D. Liang, N. Xu, B. Zhu, W. Li, P. Yao, Y. Jiang, X. Min, Z. Huang, *Sci. Adv.* **2022**, 8, 0411.

[4] S. J. Kline, *ASME J Fluids Eng.* **1985**, 107, 153.

[5] Z. M. Zhang, Luby, *Nano/microscale heat transfer*, Springer, **2007**.

[6] ANSYS Lumerical 2020 R2.4. https://www.lumerical.com/, accessed: August 18, 2023.

[7] M. Qin, H. Han, F. Xiong, Z. Shen, Y. Jin, S. Han, A. Usman, J. Zhou, R. Zou, *Adv. Funct. Mater.* **2023**, 2304073.

[8] Z. L. Wei Gao, Kai Wu, and Yongping Chen, *Adv. Funct. Mater.* **2021**, 21, 2100535.

[9] C. Feng, P. Yang, H. Liu, M. Mao, Y. Liu, T. Xue, J. Fu, T. Cheng, X. Hu, H. J. Fan, K. Liu, *Nano Energy* **2021**, 85, 105971.

[10] T. Wang, Y. Wu, L. Shi, X. Hu, M. Chen, L. Wu, *Nat. Commun.* **2021**, 12, 365.

[11] H. Zhang, K. C. S. Ly, X. Liu, Z. Chen, M. Yan, Z. Wu, X. Wang, Y. Zheng, H. Zhou, T. Fan, *Proc. Natl. Acad. Sci. U.S.A.* **2020**, 117, 14657.

[12] T. Li, Y. Zhai, S. He, W. Gan, Z. Wei, M. Heidarinejad, D. Dalgo, R. Mi, X. Zhao, J. Song, *Science* **2019**, 364, 760.

[13] L. Zhou, H. Song, J. Liang, M. Singer, M. Zhou, E. Stegenburgs, N. Zhang, C. Xu, T. Ng, Z. Yu, B. Ooi, Q. Gan, *Nat. Sustain.* **2019**, 2, 718.

[14] J. Mandal, Y. Fu, A. C. Overvig, M. Jia, K. Sun, N. N. Shi, H. Zhou, X. Xiao, N. Yu, Y. Yang, *Science* **2018**, 362, 315.
